# Elevated CO_2_ Reduces the Resistance and Tolerance of Tomato Plants to *Helicoverpa armigera* by Suppressing the JA Signaling Pathway

**DOI:** 10.1371/journal.pone.0041426

**Published:** 2012-07-19

**Authors:** Huijuan Guo, Yucheng Sun, Qin Ren, Keyan Zhu-Salzman, Le Kang, Chenzhu Wang, Chuanyou Li, Feng Ge

**Affiliations:** 1 State Key Laboratory of Integrated Management of Pest and Rodents, Institute of Zoology, Chinese Academy of Sciences, Beijing, People's Republic of China; 2 Graduate School, Chinese Academy of Sciences, Beijing, People's Republic of China; 3 Jining Normal College, Inner Mongolia Autonomous Region, Jining, People's Republic of China; 4 Department of Entomology, Texas A&M University, College Station, Texas, United States of America; 5 State Key Laboratory of Plant Genomics, National Centre for Plant Gene Research, Institute of Genetics and Developmental Biology, Chinese Academy of Sciences, Beijing, People's Republic of China; Max Planck Institute for Chemical Ecology, Germany

## Abstract

Both resistance and tolerance, which are two strategies that plants use to limit biotic stress, are affected by the abiotic environment including atmospheric CO_2_ levels. We tested the hypothesis that elevated CO_2_ would reduce resistance (i.e., the ability to prevent damage) but enhance tolerance (i.e., the ability to regrow and compensate for damage after the damage has occurred) of tomato plants to the cotton bollworm, *Helicoverpa armigera*. The results showed that elevated CO_2_ reduced resistance by decreasing the jasmonic acid (JA) level and activities of lipoxygenase, proteinase inhibitors, and polyphenol oxidase in wild-type (WT) plants infested with *H. armigera*. Consequently, the activities of total protease, trypsin-like enzymes, and weak and active alkaline trypsin-like enzymes increased in the midgut of *H. armigera* when fed on WT plants grown under elevated CO_2_. Unexpectedly, the tolerance of the WT to *H. armigera* (in terms of photosynthetic rate, activity of sucrose phosphate synthases, flower number, and plant biomass and height) was also reduced by elevated CO_2_. Under ambient CO_2_, the expression of resistance and tolerance to *H. armigera* was much greater in wild type than in *spr2* (a JA-deficient genotype) plants, but elevated CO_2_ reduced these differences of the resistance and tolerance between WT and *spr2* plants. The results suggest that the JA signaling pathway contributes to both plant resistance and tolerance to herbivorous insects and that by suppressing the JA signaling pathway, elevated CO_2_ will simultaneously reduce the resistance and tolerance of tomato plants.

## Introduction

In the last 250 years, atmospheric carbon dioxide (CO_2_) has risen from 280 ppm to greater than 390 ppm, and is anticipated to reach at least 550 ppm by year 2050 [1]. Because elevated CO_2_ increases the carbon to nitrogen (C∶N) ratio and reduces the N content in the tissue of most plant species, elevated CO_2_ is expected to alter plant synthesis of phenolics, terpenes, and other secondary metabolites [2], [3]. Such changes in C∶N and in the content of secondary metabolites will alter the nutritional quality and palatability of host plants for herbivores and could therefore affect the performance of herbivorous insects [4].

Plants have evolved a variety of mechanisms to reduce the negative impacts of herbivory [5], [6]. When damaged by herbivorous insects, plants can produce herbivore-deterrent metabolites or defensive proteins to limit the damage [7]. This kind of induced defense (i.e., resistance) is energy and resource costly, however, and cannot be maintained at high levels throughout the growing season [8]. An alternative to resistance is tolerance, which compensates for tissue loss after insect attack [9]. In expressing tolerance, plants reallocate energy and resources from undamaged to damaged tissues (for example, by increasing sucrose-transport enzymes in the damaged tissues) and increase photosynthetic rates and growth parameters [10], [11]. Although researchers generally assume that there is a trade-off between resistance and tolerance (i.e., plants with high resistance have low tolerance and vice versa), the relationship between plant resistance and tolerance to herbivores varies among studies and often depends on the plant species, soil resource, and environment [12], [13].

Elevated CO_2_ is likely to increase constitutive levels of defensive metabolites, including phenolics and tannins, in plant leaves [2], [14], and such increases in phenolics and tannins have an negative influence on the development and fitness of chewing herbivorous insects [15]. However, the induced phenolic compounds are decreased by elevated CO_2_ when responding to damage of insect [16]. Additionally, jasmonic acid (JA) signaling defense (JA is considered as the most important defense hormone involved in resistance against chewing insects) has been reported to be suppressed by elevated CO_2_ [17], and CO_2_-induced decreases in the expression of downstream genes of JA pathway (i.e., proteinase inhibitors) increased the consumption of soybean leaves by herbivorous insects [18].

Little is known about how CO_2_ affects plant tolerance to herbivores but the possible effects of resource availability on tolerance have been described by three classic models or hypotheses. The compensatory continuum hypothesis (CCH) predicts that plants growing in resource-rich or low-competition environments will be more tolerant to herbivores than those growing in resource-poor, stressful environments [19]. The main rival to the CCH is the growth rate model (GRM), which predicts that plants grow at a low relative growth rate will be more tolerant than plants grow at a high relative growth rate, because, unlike plants growing in stress-free environments, plants growing in stressful environments are not growing at their maximum rate and therefore have the potential to increase their growth rate [19]. The limiting resource model (LRM) predicts that tolerance will depend on the particular resource that is limiting plant fitness and how acquisition of that resource is affected by herbivory; according to the LRM, the relative effects of a stressful vs. a stress-free environment on tolerance will therefore depend on the nature of the resource [20].

Some researchers have reported that elevated CO_2_ increased plant susceptibility to herbivorous insects [21], [22], [23], while others found that elevated CO_2_ increased compensatory growth in response to artificial herbivory, i.e., in response to researcher removal of buds from cotton plants [24], [25]. Elevated CO_2_ could possibly affect the re-growth ability or tolerance by increasing C∶N and by decreasing the N concentration of plant tissues [26]. Although research has established that JA plays a crucial role in plant resistance to herbivorous insects [27] and that plant tolerance and resistance are not independent [13], it is still unclear how tolerance is affected by the JA signaling pathway and how the JA signaling pathway, and therefore resistance and tolerance, are affected by elevated CO_2_.

Using the cotton bollworm, *Helicoverpa armigera*, and tomato, the current study investigated the relationships between elevated CO_2_, insect herbivory, the JA pathway, and plant tolerance and resistance. *Helicoverpa armigera* is a leaf-chewing insect that causes great damage to cotton, tomato, and many other crops in Northern China [28]. To determine whether elevated CO_2_ influenced resistance and tolerance of tomato plants by affecting the JA pathway, we used the JA pathway-impaired mutant *spr2*. We tested the hypothesis that, by altering the JA signaling pathway, elevated CO_2_ would reduce tomato plant resistance (the ability to prevent damage) against *H. armigera* while enhancing plant tolerance (the ability to re-grow after *H. armigera* damage). Our specific aims were to determine (1) whether elevated CO_2_ affects the JA-dependent defense of tomato plants and the midgut enzyme activities of cotton bollworm associated with different tomato genotypes, and (2) whether elevated CO_2_ affects tolerance (in terms of re-growth ability, as indicated by photosynthetic rate, sucrose phosphate synthases, sucrose synthases, biomass, flower number, height, and branch length) of wild-type and *spr2* plants after *H. armigera* damage.

## Materials and Methods

### Open-top chambers

This experiment was carried out using eight octagonal, open-top chambers (OTC), each 4.2 m in diameter, located at the Observation Station of the Global Change Biology Group, Institute of Zoology, Chinese Academy of Science (CAS) in Xiaotangshan County, Beijing, China (40°11′N, 116°24′E). The atmospheric CO_2_ concentration treatments were: (1) current atmospheric CO_2_ levels (375 µl/L) (“ambient CO_2_”), and (2) doubled ambient CO_2_ levels (750 µl/L) (“elevated CO_2_”). Four OTCs were used for each CO_2_ concentration treatment. During the period from seedling emergence to the harvesting of tomato plants, CO_2_ concentrations were monitored and adjusted with an infrared CO_2_ analyzer (Ventostat 8102; Telaire, Goleta, CA, USA) once every 20 min to maintain the CO_2_ concentrations. The automatic-control system for adjusting the levels of CO_2_ concentration, as well as specifications for the OTC, are detailed in Chen and Ge [29].

### Tomato plants

Wild-type (WT) tomato plants (*Lycopersicum esculentum* cv. Castlemart) and jasmonate-deficient mutant plants (*spr2*) were kindly provided by Professor C. Li of the Institute of Genetics and Developmental Biology, the Chinese Academy of Sciences. The JA-biosynthesis mutant, suppressor of prosystemin-mediated responses2 (*spr2*), reduces chloroplast ω3 fatty acid desaturase, which impairs the synthesis of JA [30]. WT tomato was the parent for the *spr2* mutant. After they had grown in sterilized soil for 2 weeks, the tomato seedlings were individually transplanted into large plastic pots (20 cm diameter and 22 cm height) containing sterilized loamy field soil (organic carbon, 25 g/kg; N, 500 mg/kg; P, 200 mg/kg; K, 300 mg/kg) and placed in OTCs on 23 May 2010. Each OTC contained 40 plants. Pots were rearranged randomly within each OTC once every week. No chemical fertilizers and insecticides were used. Water was added to each pot once every 2 days.

### Herbivore treatment

**Figure 1:**
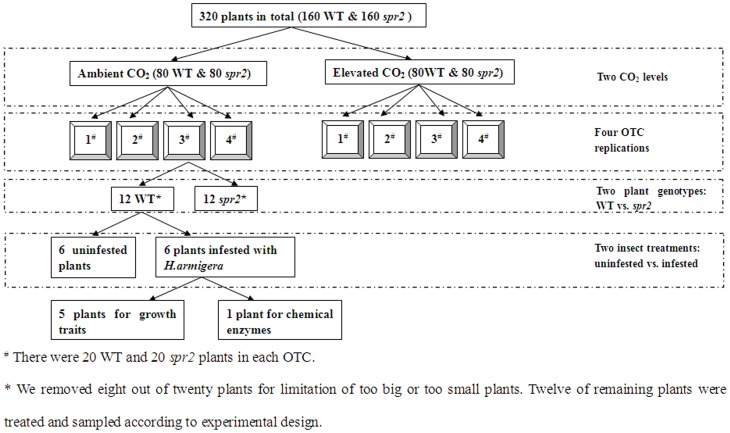
Experimental design and replications. A flow diagram of the design used for the plant treatments, tissue collection and replications. There were two CO_2_ levels, two plant genotypes, two insect treatments and four OTC replications for plant treatments.

In its native habitat in China, *Helicoverpa armigera* is one of the most abundant of the lepidopteran herbivores on *L. esculentum* cv. Castlemart. *H. armigera* larvae tend to attack leaves and fruits. Thirty-six days after the tomato plants had been placed in the OTCs, 40 plants of each genotype at each CO_2_ level ( = 10 plants per OTC) were randomly selected and infested with *H. armigera*. Six 4^th^-stage *H. armigera* larvae were placed on each of two leaves at mid-plant height, and the leaves were caged (80 mesh gauze); corresponding leaves of control plants were caged in the same way. After 2 days, the larvae were removed and were weighed and analyzed for midgut proteases as described later in the Methods. The leaves of eight randomly selected plants infested with larvae and of eight noninfested plants were harvested and immediately stored in liquid nitrogen (Fig. 1).

### Plant photosynthesis

Net photosynthetic rate was determined in the presence or absence of herbivores on four 5-week-old plants for each genotype at each CO_2_ level. Gas exchange was measured on the distal portion of the leaf blade of undamaged leaves using a Li-6400 with a red/blue LED light source (6400-02B; Li-Cor, Lincoln, NE, USA). The CO_2_ concentration of the incoming air was adjusted to 400 *µ*mol mol^−1^ CO_2_ or 750 *µ*mol mol^−1^. Relative humidity corresponded to ambient conditions. Before gas exchange was measured, photosynthetic active radiation (PAR) for the leaf in the measuring cuvette was increased in steps to 1200 *µ*mol m^−2^ s^−1^.When the CO_2_ assimilation rate was stable for at least 2 min, a light response curve was recorded. Measurements were carried out after herbivores had been allowed to feed on plants for 2 days.

### JA measurement

A 0.5 g sample of fresh leaves was ground to a fine powder on ice. The powder was mixed with 4 ml of 80% methanol (80 methanol/20 water, V/V) and kept at −20°C for 12 h, and then added to 6 *µ*l of [9], [10]-dihydro-JA (dihydro-JA) for use as an internal standard. The total extracted preparation was centrifuged at 8,000 *g* for 20 min. The condensed endogenous JA were extracted according to procedures described by ren *et al.*, [31].

Endogenous JA and its internal standards (dihydro-JA) were analyzed using full GC/MS scans. Retention times were identified using Xcalibur 1.2, the NIST 2003 mass library. Endogenous JA was measured by GC-MS selected ion monitoring (SIM). The characteristic ions (m/z) were 151/224 for JA and 153/226 for the internal standard (dihydro- JA).

### Plant enzyme activity (SPS, SS, LOX, PIs, PPO, POD, and PAL)

For analyses of sucrose synthases (SS) and sucrose phosphate synthases (SPS), 0.5 g of fresh tomato leaves was homogenized for 1.5 min at 4°C in 1∶10 (fresh weight/buffer volume ratio) 100 mMphosphate buffer, pH 7.4, containing 100 Mm KCl and 1 mM EDTA. Homogenates were centrifuged at 10 000 *g* for 10 min, and the supernatants were subjected to SS and SPS analysis. SS and SPS were analyzed according to the protocol of the reagent kit (Nanjing Jiancheng Bioengineering Institute, Nanjing, Jiangsu Province, China).

About 0.1 g of frozen leaf tissue was used for determination of plant defensive enzyme activity, including the activities of lipoxygenase (LOX), proteinase inhibitors (PIs), polyphenol oxidase (PPO), peroxidase (POD), and phenylalanine ammonia lyase (PAL). Extract was obtained from individual leaflets by grinding them in a 50 mM Tris HCl buffer (pH 7.8, 3 ml/g of leaf tissue) containing 7% polyvinylpolypyrrolidine, 1.67 mM phenylthiourea, 0.3 M KCl, and 0.4 mM ascorbic acid. This extract was immediately frozen for later use. For assays, the thawed extract was centrifuged at 13,000 *g* for 10 min, and enzyme activity was measured in the supernatant. LOX assays were performed in a 1-ml reaction mixture containing 20 *µ*l of supernatant and 980 *µ*l of reaction buffer. The reaction buffer consisted of 0.113 g of linoleic acid, which had been dissolved in 3 ml of methanol in 100 ml pH 7.8 Tris-HCl buffer (w/v, pH 7.0), and 800 µl of Tween-20. PIs were measured as in Thaler *et al.* [32]. PPO activity was measured as in Mahanta *et al.* [33]. POD activity was determined according to Tegelberg *et al.* [34]. Reaction mixtures for determination of PAL activity contained 950 *µ*l of 0.02 mM L-phenylalanine (0.33 g of L-phenylalanine dissolved in 100 mL of Tris-HCl, pH 7.8) and 50 µl of supernatant. The changes in absorbance were detected with a spectrophotometer at 290 nm (SPEKTRAmax®Plus; Molecular Devices, Sunnyvale, California, USA).

### Midgut proteases of *H. armigera*

After they had fed on plants for 48 h, 30 larvae of uniform size from each treatment were selected. Larvae were anaesthetized by chilling on ice, and their midguts were removed. The midgut tissue was cut longitudinally, and the external surface of the midgut tissue was cleaned by placing it in 0.15 mol/L NaCl. Finally, the tissues were stored at −80°C.

All enzyme substrates were obtained from Sigma-Aldrich. Weak alkaline trypsin-like enzyme (WATE) activity with Nα-tosil-L-arginin methyl ester (TAME) substrates was assayed spectrophotometrically at 248 nm according to the methods of Erlanger *et al.* [35]. The substrates were initially dissolved in dimethyl formamide (DMF) and further dissolved in 20 mM Tris–HCl buffer, pH 8.5.

Active alkaline trypsin-like enzyme (AATE) activity was measured according to Erlanger *et al.* [35] by using N-benzoyl-DL-arginin-ρ-nitroanilide (BApNA) and 7.5% (v/v) dimethyl sulfoxide (DMSO) dissolved in Glycine-NaOH buffer (0.1 mM, pH 10.5) at a final concentration of 1.4 mM. The mixtures were incubated for 20 min at 30°C, and the reactions were stopped by adding 0.5 ml of 30%(v/v) acetic acid. The enzyme activity was measured at 406 nm.

Chymotrypsin activity in extracts was determined spectrophotometrically according to Blackwood *et al.* [36], based on the hydrolysis of N-benzoyl-L-tyrosin ethyl ester (BTEE), which was determined at 256 nm (l M in 10% (v/v) methanol with 0.15 M NaC1). Tris-HCl buffer (0.2 M, pH 8.5) was used. One unit of enzyme activity was defined as 1 mol of BTEE hydrolyzed per min. The extinction coefficient of BTEE was 964.

Total protease was measured with azocasein as the substrate. Azocasein (20 mg ml^−1^) was dissolved in 0.15 M NaC1. The sample was then dissolved in 0.2 M glycine-NaOH buffer (pH 8.0). The reaction was run by adding 300 *µ*l of reaction buffer to 0.3 ml of the azocasein solution and incubating the mixture for 24 h at 30°C. The reaction was stopped by adding 0.6 ml of 20% (W/V) trichloroacetic acid (TCA). Samples from each time point were centrifuged at 10000 *g* for 15 min at 4°C, and the absorbance of the supernatant was measured once at 366 nm. One absorbance unit from the mixture was defined as one unit of azocasein under the given assay conditions.

### Mean relative growth rate of *H. armigera*

*H. armigera* larvae were weighed with an automatic electrobalance before and after they had fed on tomato plants. Mean relative growth rate (MRGR) was calculated following Chen *et al.* [37]: MRGR = (ln W_2_−ln W_1_)/t, where W_1_ is the initial weight; W_2_ is the final weight; and t is the time in days between weighings. For each combination of genotype and CO_2_ level, 85 larvae were weighed.

### Resistance and tolerance analysis

The following variables were considered to be measures of resistance: the activities of LOX, PIs, PPO, POD, and PAL in plants; the activities of midgut enzymes in *H. armigera*; and the MRGR of *H. armigera*.

Tolerance was measured by comparing the parameters on plants that were or were not exposed to *H. armigera* and that were exposed to ambient or elevated CO_2_. In the short term, the following variables were considered to be measures of tolerance: photosynthetic rate, sucrose phosphate synthases and sucrose phosphate synthases. In the long term, the following variables were considered to be measures of tolerance (i.e. re-growth ability): total branch length per plant, plant height, cumulative number of flowers per plant, and final root and shoot mass. These data were obtained 60 days after the larvae had been added from five plants from each combination of genotype and herbivore treatment per OTC ( = 20 plants per OTC and 160 plants in total).

JA content was considered to be one measure of both tolerance and resistance.

### Statistical analysis

A split-split plot design was used to analyze the univariate responses of the measured variables (i.e., plant traits, chemical components, phytohormones) (ANOVA, PASW, 2009). In the following ANOVA model, CO_2_ and block (a pair of OTCs with ambient and elevated CO_2_) were the main effects, tomato genotype was the subplot effect, and herbivore level was the sub-subplot effect:
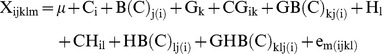
where C is the CO_2_ treatment (i = 2), B is the block (j = 4), G is the tomato genotype (k = 2), and H is the herbivore treatment (l = 2). X_ijklm_ represents the error because of the smaller scale differences between samples and variability within blocks (ANOVA, SAS institute, 1996). Effects were considered significant if P<0.05. The effect of block and the interactive effects of block and other factors were not significant (P>0.45), and the effect of block and its interaction with other factors are not presented so as to simplify the presentation. Least significant difference (LSD) tests were used to separate means when ANOVAs were significant. For quantifying the midgut protease and weight of *H. armigera* on different tomato genotypes under two CO_2_ levels, a split-plot design was also applied, with CO_2_ and block as the main effects and tomato genotype as the subplot effect. R software (version 2.15.0, http://www.r-project.org/) was used to calculate the standard error of the difference value of parameters between herbivore infested and uninfested treatment.

## Results

### Photosynthesis and growth

**Figure 2:**
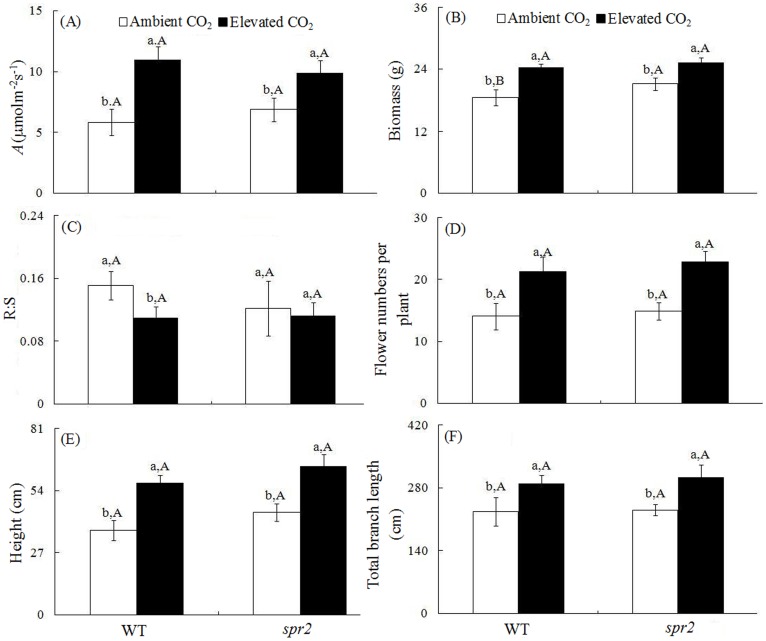
Growth parameters of two tomato genotypes grown under ambient and elevated CO_2_ without *H. armigera* infestation. (A) Photosynthetic rate (*A*), (B) biomass, (C) root biomass: shoot biomass ratio (R∶S), (D) cumulative flower number, (E) plant height, and (F) total branch length. Each value represents the average (±SE) of 20 replicates. Different lowercase letters indicate significant differences between CO_2_ level within the same tomato genotype (LSD test: d.f. = 3.12; *P*<0.05). Different uppercase letters indicate significant differences between WT plants and *spr2* plants within the same CO_2_ level (LSD test: d.f. = 2.9; *P*<0.05).

Relative to ambient CO_2_ and in the absence of *H. armigera*, under elevated CO_2_, the photosynthetic rate were significantly increased by 87.9% and 43.8%, biomass by 31.6% and 19.8%, flower number by 51.1% and 53.0%, total branch length by 27.5% and 31.8%, and plant height by 56.7% and 44.5% for WT and *spr2* plants, respectively. The root to shoot ratio (R∶S) were significantly decreased by 27.2% for WT plants and by 8.2% for *spr2* plants under elevated CO_2_ (Fig. 2).

### SPS and SS activity

**Figure 3:**
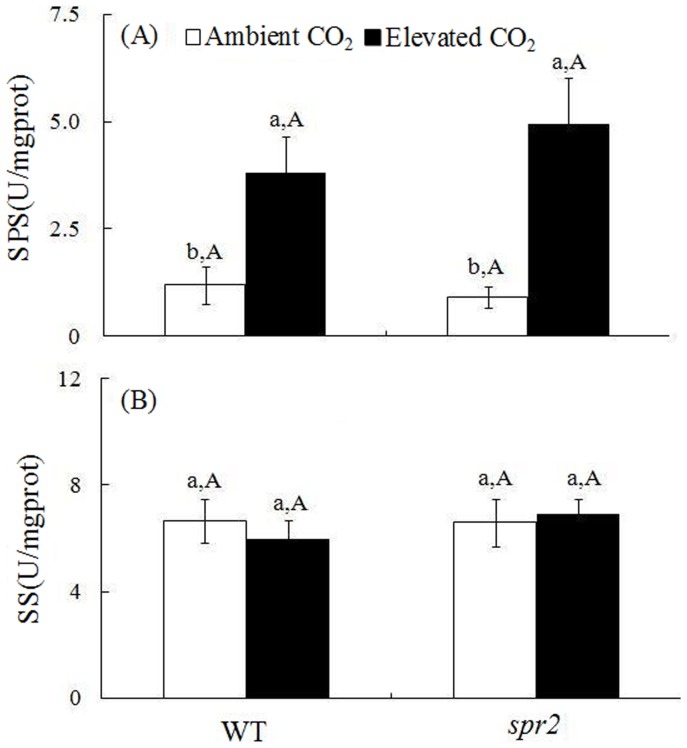
Activity of sugar transport enzymes in leaves of two tomato genotypes grown under ambient and elevated CO_2_ without *H. armigera*. (A) sucrose phosphate synthase (SPS) activity, and (B) sucrose synthase (SS) activity. Each value represents the average (±SE) of four replicates. Different lowercase letters indicate significant differences between CO_2_ level within the same tomato genotype (LSD test: d.f. = 3.12; *P*<0.05). Different uppercase letters indicate significant differences between WT plants and *spr2* plants within the same CO_2_ level (LSD test: d.f. = 2.9; *P*<0.05).

Relative to ambient CO_2_ and in the absence of *H. armigera*, SPS activity were significantly increased by 2.2-fold for WT plants and by 4.4-fold for *spr2* plants under elevated CO_2_ (Fig. 3A) but SS activity of either WT or *spr2* plants were not affected (Fig. 3B). SPS and SS activities were unaffected by genotype (Fig. 3).

### JA level and defense enzyme activity

**Figure 4:**
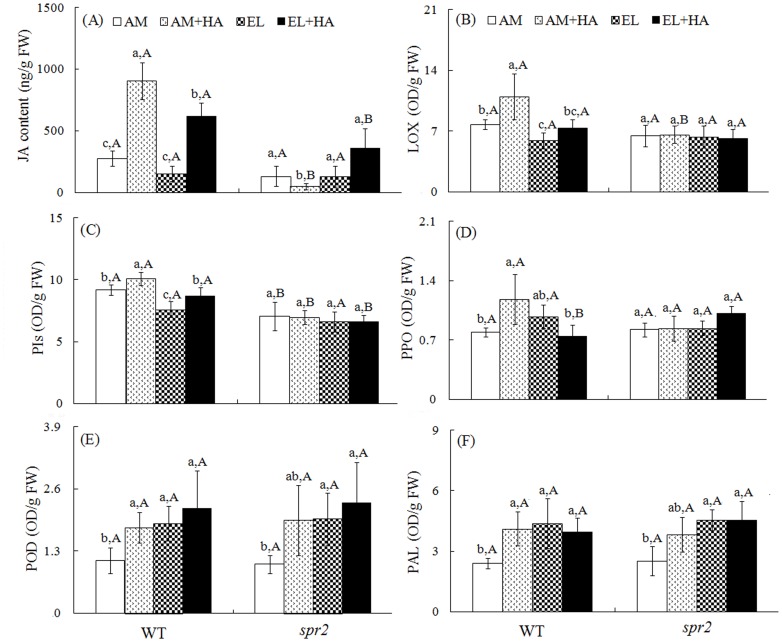
Chemical defensive components in two tomato genotypes grown under ambient (AM) and elevated CO_2_ (EL) without and with *H. Armigera* (+HA). (A) JA content, and the activity of (B) lipoxygenase (LOX), (C) proteinase inhibitors (PIs), (D) polyphenol oxidase (PPO), (E) peroxidase (POD), and (F) phenylalanine ammonia lyase (PAL). Each value represents the average (±SE) of four replicates. Different lowercase letters indicate significant differences among combinations of *H. armigera* and CO_2_ level within the same tomato genotype (LSD test: d.f. = 3.12; *P*<0.05). Different uppercase letters indicate significant differences between WT plants and *spr2* plants within the same CO_2_ (LSD test: d.f. = 2.9; *P*<0.05).

Genotype, *H.armigera* infestation, the interaction between CO_2_ and genotype, the interaction between genotype and *H.armigera* infestation, as well as the interaction among CO_2_ level, genotype and *H.armigera* infestation, significantly affected plant JA content (Table S1). JA levels and LOX, PI, and PPO activities of WT plants were decreased when damaged by *H. Armigera* under elevated CO_2_ (Fig. 4A–D). Elevated CO_2_ also decreased LOX and PI activity of undamaged WT plants. POD and PAL activity of undamaged plants in both genotypes were higher under elevated CO_2_ (Fig. 4E, F).

*H. armigera* significantly increased the levels of JA and all defensive proteins in WT plants under ambient CO_2_ (Table S1, Fig. 4). Under elevated CO_2_, in contrast, *H. armigera* only increased JA levels and PI activity in WT plants.

### Midgut proteases and weight of *H. armigera*

**Figure 5:**
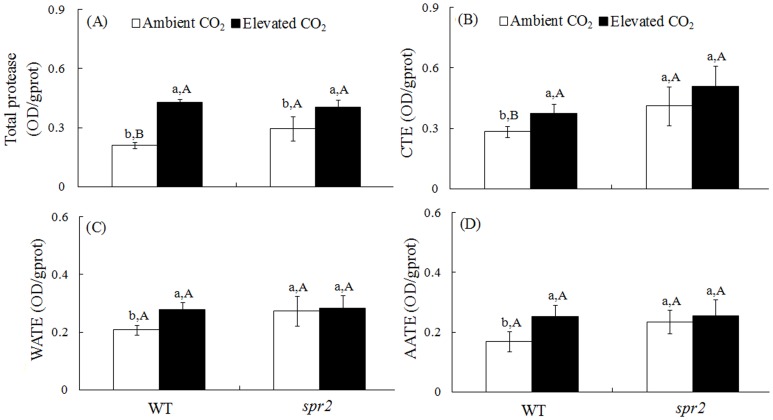
Activities of digestive proteases in the guts of *H. armigera* that fed for 2 days on tomato genotypes grown under ambient and elevated CO_2_. (A) total protease, (B) chymotrypsin (CTE) activity, (C) weak alkaline trypsin-like enzyme (WATE) activity, and (D) active alkaline trypsin-like enzyme (AATE) activity. Each value represents the average (±SE) of four replicates. Different lowercase letters indicate significant differences between ambient CO_2_ and elevated CO_2_ treatment. Different uppercase letters indicate significant differences between feeding on the WT plants and *spr2* plants within the same CO_2_.

**Figure 6:**
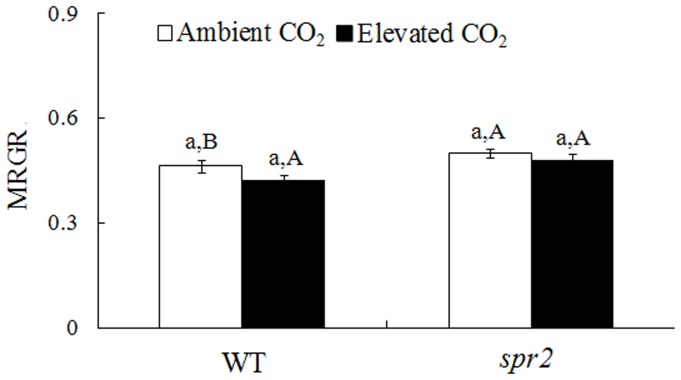
Mean relative growth rate (MRGR) of *H. armigera* that fed for 2 days on tomato genotypes grown under ambient and elevated CO_2_. Each value represents the average (±SE) of 85 replicates. Different lowercase letters indicate significant differences between ambient CO_2_ and elevated CO_2_ treatment. Different uppercase letters indicate significant differences between feeding on WT plants and *spr2* plants within the same CO_2_ treatment.

Total Proteolysis was affected by CO_2_ and the interaction between CO_2_ and genotype (Table S2). *H. armigera* that consumed leaves of WT plants grown under elevated CO_2_ had substantially higher gut protease activities than those that consumed leaves of WT plants grown under ambient CO_2_ (Table S2, Fig. 5). However, elevated CO_2_ did not affect gut protease activity in *H. armigera* that consumed *spr2* foliage. Under ambient CO_2_, *H. armigera* that consumed leaves of WT plants had higher total protease and CTE activity than those that consumed *spr2* foliage (Fig. 5A, B). Moreover, *H. armigera* that consumed leaves of WT plants had a lower MRGR than those that consumed *spr2* foliage under ambient CO_2_ (Fig. 6).

### Regrowth and sucrose transportation after *H. armigera* attack

**Figure 7:**
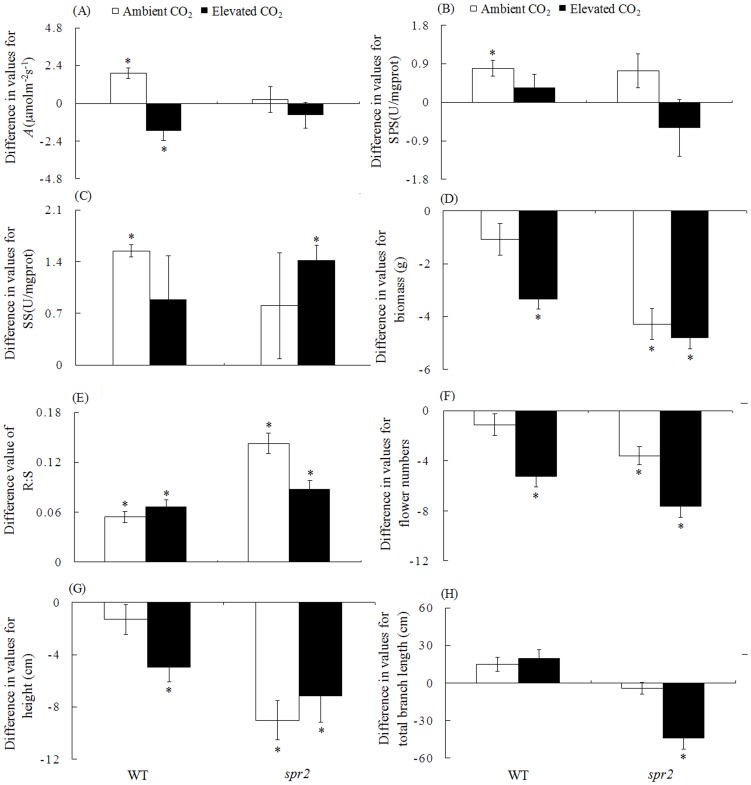
Tolerance (as indicated by differences in values of growth traits or enzyme activities between damaged plants and undamaged plants) of two tomato genotypes grown under ambient and elevated CO_2_. Damaged plants are those that were fed on by *H. armigera*. (A) photosynthetic rate (*A*), (B) sucrose phosphate synthase (SPS), (C) sucrose synthase (SS), (D) biomass, (E) root biomass: shoot biomass (R∶S), (F) cumulative flower number, (G) plant height, and (H) total branch length. Each value represents the average (±SE) of 20 replicates. Symbols above columns indicate levels of significant differences between variables of damaged and undamaged plants (*, P<0.05). Positive differences indicate that values were greater for damaged plants than for undamaged plants.

For WT plants under ambient CO_2_, *H. armigera* increased the photosynthetic rate and SPS and SS activities but did not significantly affect biomass, flower number, height, and total branch length. For WT plants under elevated CO_2_, however, *H. armigera* not only reduced the photosynthetic rate but also decreased plant biomass by 13.7% and flower number by 24.9% (Fig. 7D, F). For *spr2* plants under ambient CO_2_, *H. armigera* reduced plant biomass by 20.1%, flower number by 24.2%, and plant height by 11.6% (Fig. 7D, F, G). For *spr2* plants under elevated CO_2_, *H. armigera* reduced plant biomass by 18.8%, flower number by 33.8%, and total branch length by 14.6% (Fig. 7D, F, H). In addition, *H. armigera* increased the R∶S of both genotypes under both CO_2_ levels (Fig. 7E).

## Discussion

### The effects of elevated CO_2_ on resistance and tolerance ability of tomato plants to *H.armigera* attack

Many studies have evaluated the relationship between plant resistance and tolerance to herbivores [38], [39], but little information is available regarding how the relationship between tolerance and resistance is affected by an abiotic stress such as global CO_2_ enrichment. Our results suggested that elevated CO_2_ decreased tomato plant resistance against *H. armigera* by suppressing the critical defensive signal molecule JA and JA-pathway-related defensive enzymes. Our results also indicated that tomato plants grown under elevated CO_2_ are less tolerant to *H. armigera* than plants grown under ambient CO_2_. Phenotypic plasticity is a principal means by which plants cope with biotic or abiotic stress [40], and the decreased resistance and tolerance to herbivores under elevated CO_2_ in this study suggests that elevated CO_2_ reduces the phenotypic plasticity of plant response to herbivorous insect attack.

Prior studies have revealed that elevated CO_2_ increases total nonstructural carbohydrates in plant tissues and that the excess C is probably allocated to the increased synthesis of secondary metabolites, such as terpenes and phenolics [41], [42], which in turn can reduce the development of chewing insects [43]. Furthermore, PAL is known to be a principle enzyme involved in a rate-limiting step of phenolic biosynthetic process [41]. Our results with both genotypes of tomato plants were consistent with the previous finding that elevated CO_2_ increases PAL activity in plants (Fig. 4F). Generally, attack by chewing insects induces a complex set of defense responses in plants [44]. In WT plants under ambient CO_2_, *H. armigera* attack increased anti-oxidant enzymes (in terms of POD), followed by triggered JA signaling pathway defense (in terms of JA and LOX) and caused plants to increase PIs and PPO activities (Fig. 4A–E). To the undamaged WT plants, elevated CO_2_ decreased LOX and PIs activity. Additionally, when damaged by *H.armigera*, elevated CO_2_ reduced LOX activity and JA level as well as PI and PPO activity in WT plants (Fig. 4A–D). It seems that elevated CO_2_ tends to impair the JA-dependent defense induced by *H. armigera*. Although responses to elevated CO_2_ by plants and insects are species-specific [14], the current results are not the first to show that elevated CO_2_ changes the plant–insect interaction by modifying the JA-dependent pathway. In soybean plants, elevated CO_2_ also suppressed the JA signaling pathway and increased susceptibility to the Japanese beetle, *Popillia japonica* [17], [18].

Proteinase inhibitors (PIs) of plants are able to reduce the feeding fitness of chewing insects by suppressing insect gut proteases [45]. WT plants grown under elevated CO_2_ had reduced PI activity, the reduced resistance resulted in increased gut protease activities for *H. armigera* (Fig. 5); these results may explain our previous finding that *H. armigera* consumed more wheat biomass when the wheat was grown under elevated vs. ambient CO_2_ conditions [46]. Although increases in gut protease activities may result in increased consumption under elevated CO_2_, the *H. armigera* MRGR did not change (Fig. 6). Perhaps the increased consumption only complemented feeding and enabled the insect to maintain development and growth when consuming leaves grown under elevated CO_2_, i.e., on leaves with a reduced N concentration.

Plant tolerance to herbivorous insects can depend on the availability of particular resources such as C resources [9]. Elevated CO_2_ increases C assimilation and causes re-allocation of C (especially sucrose) in plant tissue [47]. In the transport of sucrose from leaves to sink tissues via phloem, SPS and SS are key regulatory enzymes [48]. Because elevated CO_2_ significantly increases plant growth and C metabolism (Fig. 2), the CCH hypothesis would predict that plant tolerance to herbivores would be increased in the resource-rich, elevated-CO_2_ environment. The CCH hypothesis, however, was not supported by the current study. Under ambient CO_2_, WT plants expressed substantial tolerance to *H. armigera* attack in that herbivory increased C assimilation and sucrose synthesis (as indicated by SPS activity in leaves) and transportation (as indicated by SS activity in roots) such that plant biomass and other parameters measured at week 8 did not differ between plants with and without *H. armigera* infestation. This suggests that WT plants can completely compensate for *H. armigera* damage under ambient CO_2_. Under elevated CO_2_, in contrast, *H. armigera* consumption of WT plants reduced photosynthesis, biomass, flower number, and plant height and did not increase SPS and SS activity. We conclude that elevated CO_2_ reduces the tolerance of WT tomato plants to *H. armigera*. Furthermore, our results are consistent with the GRM hypothesis, which predicts that plants growing under conditions that promote a high growth rate will be less tolerant to herbivores than plant growing under conditions that reduce the growth rate [23].

### The interaction between elevated CO_2_ and jasmonate signalling

In addition to the essential role of the JA pathway in resistance against herbivorous insects, JA has been found to regulate the interaction between tolerance and resistance in *Nicotiana attenuata* against *Manduca sexta* [49]. While WT plants in the current study exhibited substantial tolerance against *H. armigera* under ambient CO_2_, *spr2* mutant plants did not, i.e., tolerance under ambient CO_2_ was much lower in *spr2* than in the WT (Fig. 7). This is consistent with that finding that treatment of *Populus* with JA increased C transport to the roots, nutrient uptake, and regrowth capacity, and therefore increased tolerance [50]. Results concerning the effect of the JA pathway on tolerance, however, have been inconsistent. *N. attenuata* and its JA-deficient genotype (*asLOX*) did not differ in capsule production after simulated herbivore attack [9], and the tolerance to defoliation did not differ between WT *Arabidopsis* and the overexpressing-JA genotype JMT [51]. This indicates that tolerance to herbivorous insects may depend on some mechanisms other than the JA pathway. Under elevated CO_2_, the tolerance of *spr2* plants to *H. armigera* was not lower than WT plants except the total branch length (Fig. 7A–G). This result confirmed that JA pathway plays an important role in tolerance of plants when attacked by *H.armigera* under elevated CO_2_. Our study suggests that the suppression of JA pathway may be one of reasons why elevated CO_2_ decreased both resistance and tolerance of tomato when damaged by *H. armigera*.

### Conclusion

**Figure 8:**
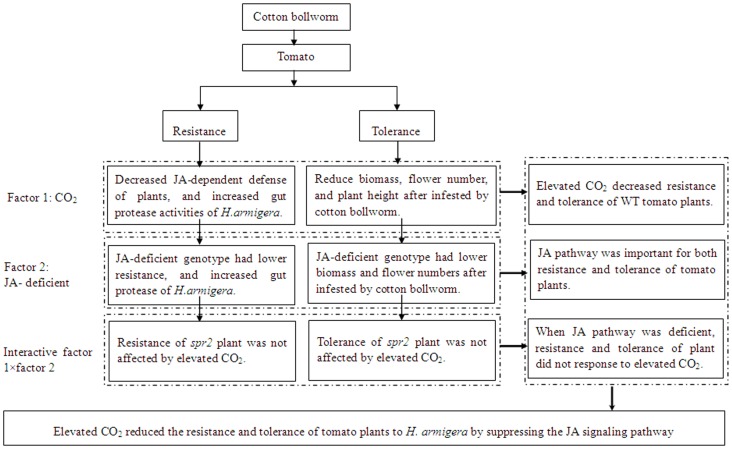
The major results and conclusion of this study were summarized. Elevated CO_2_ decreased resistance and tolerance of WT to *H. armigera*. In contrast, the resistance and tolerance of *spr2* were not changed by elevated CO_2_. Elevated CO_2_ reduces the resistance and tolerance of WT plants by suppressing the JA signaling pathway.

This study has generated a number of significant findings. First, the results support the view that the JA signaling pathway is important in both resistance and tolerance to chewing insects. Second, the results are consistent with the GRM model, which predicts that tolerance will be greater in resource-limited than in resource-unlimited environments. Third, a trade-off between resistance and tolerance as predicted by classical theory was not evident in our study [52]. Finally and perhaps most importantly, the results suggest that plants may suffer greater damage from herbivorous insects if levels of atmospheric CO_2_ continue to increase (Fig. 8).

## Supporting Information

Table S1 ***F*** **and** ***P*** **values from MANOVAs for the effect of CO_2_ level, tomato genotype and** ***H. armigera*** **infestation on growth traits, photosynthesis, and foliar chemical components of two tomato genotypes.**
(DOC)

Table S2 ***F*** **and** ***P*** **values from two-way ANOVAs for the effect of CO_2_ level and tomato genotype on MRGR and midgut proteolytic enzymes of** ***H. armigera*** **.**
(DOC)
