# Supplementary material for: Elevated CO2 Reduces the Resistance and Tolerance of Tomato Plants to Helicoverpa armigera by Suppressing the JA Signaling Pathway
Source: PLoS One. 2012 Jul 19;7(7):e41426. doi: 10.1371/journal.pone.0041426 (PMC3400665; doi:10.1371/journal.pone.0041426)
Supplement: Table S1 — F and P values from MANOVAs for the effect of CO2 level, tomato genotype and H. armigera infestation on growth traits, photosynthesis, and foliar chemical components of two tomato genotypes. (DOC) [file pone.0041426.s001.doc]

**Appendix Table**

**Supporting information**

**Table S1**. *F* and *P* values from MANOVAs for the effect of CO_2_ level, tomato genotype and *H. armigera* infestation on growth traits, photosynthesis, and foliar chemical components of two tomato genotypes.

| Plant response | Factors | CO_2_^a^ | | Gen^b^ | | HA^c^ | | CO_2_×Gen | | CO_2_×HA | | Gen×HA | | CO_2_×Gen×HA | |
| --- | --- | --- | --- | --- | --- | --- | --- | --- | --- | --- | --- | --- | --- | --- | --- |
|  |  | *F* value | *P* value | *F* value | *P* value | *F* value | *P* value | *F* value | *P* value | *F* value | *P* value | *F* value | *P* value | *F* value | *P* value |
| Growth traits | Height | 74.487 | 0.013* | 22.741 | <0.001*** | 27.937 | <0.001*** | 4.682 | 0.388 | 0.775 | 0.657 | 5.409 | 0.023* | 1.669 | 0.201 |
|  | Biomass | 80.482 | 0.012* | 6.435 | 0.013* | 159.089 | <0.001*** | 3.247 | 0.269 | 8.326 | 0.005** | 20.680 | <0.001*** | 11.116 | 0.216 |
|  | R:S^d^ | 25.578 | 0.037* | 6.894 | 0.010* | 271.320 | <0.001*** | 0.043 | 0.837 | 4.039 | 0.048* | 26.883 | <0.001*** | 9.750 | 0.002** |
|  | Flowers | 213.160 | 0.005** | 0.036 | 0.851 | 124.262 | <0.001*** | 0.892 | 0.348 | 27.324 | <0.001*** | 9.523 | 0.003** | 0.004 | 0.950 |
|  | Branch length | 35.258 | 0.027* | 6.805 | 0.011* | 0.503 | 0.480 | 1.532 | 0.219 | 3.411 | 0.069 | 18.842 | 0.000*** | 5.512 | 0.022* |
| Photo-synthesis | Photo-synthetic rate | 202.722 | <0.001*** | 0.190 | 0.668 | 0.061 | 0.807 | 0.911 | 0.352 | 8.745 | 0.008 | 0.181 | 0.675 | 2.885 | 0.107 |
| Defensive  enzymes/  hormones | PAL^e^ | 14.069 | 0.010* | 0.313 | 0.583 | 5.602 | 0.029* | 0.718 | 0.408 | 9.626 | 0.006** | 0.000 | 0.985 | 0.573 | 0.459 |
|  | PPO^f^ | 0.360 | 0.571 | 0.597 | 0.450 | 2.438 | 0.136 | 3.915 | 0.063 | 4.011 | 0.060 | 0.024 | 0.879 | 11.674 | 0.003** |
|  | LOX^g^ | 6.370 | 0.045* | 14.725 | 0.001** | 7.752 | 0.012* | 8.459 | 0.009** | 1.397 | 0.253 | 7.801 | 0.681 | 0.681 | 0.420 |
|  | POD^h^ | 5.252 | 0.062 | 0.183 | 0.673 | 11.937 | 0.003** | 0.035 | 0.854 | 2.159 | 0.159 | 0.134 | 0.721 | 0.119 | 0.734 |
|  | PIS^i^ | 12.239 | 0.013* | 76.708 | <0.001*** | 4.617 | 0.046* | 5.253 | 0.034* | 0.136 | 0.717 | 4.854 | 0.041* | 0.013 | 0.912 |
|  | JA^j^ | 1.419 | 0.278 | 72.711 | <0.001*** | 68.616 | <0.001*** | 9.933 | 0.006** | 0.398 | 0.536 | 39.937 | <0.001*** | 22.527 | <0.001*** |
| [Sucrose](app:ds:sucrose)  enzymes | SS^k^ | 0.547 | 0.487 | 2.125 | 0.162 | 21.563 | <0.001*** | 13.287 | 0.002** | 0.016 | 0.902 | 0.002 | 0.967 | 1.315 | 0.267 |
|  | SPS^l^ | 69.438 | <0.001*** | 0.705 | 0.412 | 2.467 | 0.134 | 5.687 | 0.028* | 4.919 | 0.040* | 1.540 | 0.231 | 1.174 | 0.293 |

^a^ Ambient CO_2_ vs. elevated CO_2_. ^b^ Two genotypes of tomato (Wt and *spr2*). ^c^ Infected or not infected with *H. armigera*. ^d^ Root biomass: Shoot biomass ratio. ^e^ Phenylanlanine ammonialyase. ^f^ Polyphenol oxidase. ^g^ Lipoxygenase. ^h^ Peroxidase. ^i^ Proteinase inhibitors. ^j^ Jasmonic acid. ^k^ Sucrose synthase. ^l^ Sucrose phosphate synthase. *<0.05, **<0.01, ***<0.001.
