# Supplementary material for: Elevated CO2 Reduces the Resistance and Tolerance of Tomato Plants to Helicoverpa armigera by Suppressing the JA Signaling Pathway
Source: PLoS One. 2012 Jul 19;7(7):e41426. doi: 10.1371/journal.pone.0041426 (PMC3400665; doi:10.1371/journal.pone.0041426)
Supplement: Table S2 — F and P values from two-way ANOVAs for the effect of CO2 level and tomato genotype on MRGR and midgut proteolytic enzymes of H. armigera . (DOC) [file pone.0041426.s002.doc]

**Table S2**. *F* and *P* values from two-way ANOVAs for the effect of CO_2_ level and tomato genotype on MRGR and midgut proteolytic enzymes of *H. armigera*.

| Insects responses | CO_2_^a^ | | Gen^b^ | | CO_2_×Gen | |
| --- | --- | --- | --- | --- | --- | --- |
|  | *F* value | *P* value | *F* value | *P* Value | *F* value | *P* value |
| MRGR^c^ | 4.262 | 0.040* | 15.891 | <0.001*** | 1.508 | 0.221 |
| TP^d^ | 76.649 | <0.001*** | 2.826 | 0.116 | 8.524 | 0.013* |
| CTE^e^ | 6.543 | 0.025* | 12.166 | 0.004** | 0.009 | 0.924 |
| AATE^f^ | 6.073 | 0.030* | 2.632 | 0.131 | 2.255 | 0.159 |
| WATE^g^ | 4.925 | 0.046* | 3.633 | 0.081 | 2.749 | 0.123 |

^a^ Ambient CO_2_ vs. elevated CO_2_. ^b^ Two genotypes of tomato (Wt and *spr2*). ^c^ Mean relative growth rate. ^d^ Total proteolytic enzyme. ^e^ Trypsin-like enzymes. ^f^ Active alkaline trypsin-like enzyme. ^g^ Weak alkaline trypsin-like enzyme. *<0.05, **<0.01, ***<0.001.
